# The efficacy, acceptability, and safety of five atypical antipsychotics in patients with first-episode drug-naïve schizophrenia: a randomized comparative trial

**DOI:** 10.1186/s12991-017-0170-2

**Published:** 2017-12-22

**Authors:** Congjie Wang, Wenjie Shi, Chengbing Huang, Jiannan Zhu, Wenzhong Huang, Gang Chen

**Affiliations:** 1 Department of Psychiatry, Huaian No. 3 People’s Hospital, and Teaching Hospital of Xuzhou Medical University, No. 272 West Huaihai Road, Huai’an, Zip code: 223001 Jiangsu China; 2 Psycological Department, Huaian No. 3 People’s Hospital, and Teaching Hospital of Xuzhou Medical University, No. 272 West Huaihai Road, Huai’an, Zip code: 223001 Jiangsu China

**Keywords:** Schizophrenia, Atypical antipsychotics, Efficacy, Acceptability, Safety

## Abstract

**Background:**

Differences in effectiveness and tolerability between different atypical antipsychotics may affect schizophrenic patients’ treatment adherence or prognosis. However, which kind of antipsychotic was more effective and safe in the treatment of schizophrenia is still being debated. This study attempted to understand whether there are any differences in efficacy, acceptability, and safety between the five atypical antipsychotics in patients with first-episode schizophrenia.

**Methods:**

Two hundred cases of inpatients with first-episode drug-naïve schizophrenia were randomly assigned to 6–8 weeks of treatment with either of aripiprazole, risperidone, quetiapine, olanzapine, or ziprasidone from October 2012 to November 2014. The efficacy, acceptability, and safety measurement after 6–8 weeks of treatment of the five kinds of antipsychotics were evaluated by the deduction rate of Brief Psychiatric Rating Scale (BPRS) total score, the proportion of treatment discontinuation, and adverse events, respectively. Whether the treatment discontinuation or combination therapy for baseline antipsychotics after titration mainly depended on ineffective or less effective on an initial-assigned antipsychotic during the study period.

**Results:**

BPRS total scores in each antipsychotic group were significantly decreased at the end of the study (*P* < 0.01), and only the deduction rate of BPRS total scores in the risperidone group was markedly higher than those in the groups of aripiprazole (*P* < 0.01) and olanzapine (*P* < 0.05) after controlling the impact of the differences of age of onset. There were significant differences between quetiapine (*χ* ^2^ = 5.46, *P* = 0.019), olanzapine (*χ* ^2^ = 5.6, *P* = 0.018), and ziprasidone regarding the proportion of maintaining on initially allocated therapy. In addition, the difference in treatment discontinuation between male and female patients was also significant (*χ* ^2^ = 9.897, *P* = 0.002), and odds ratio of treatment discontinuation in male and female patients was 0.37 (95% CI 0.198–0.693); however, no difference in treatment discontinuation was found between five antipsychotics. Extrapyramidal symptoms in the groups of quetiapine and olanzapine were notably less than the other three kinds of antipsychotics (*P* < 0.05), but there were no significant differences in other adverse events between the five antipsychotic groups.

**Conclusions:**

Risperidone was more effective than aripiprazole and olanzapine in treating first-episode schizophrenia. The present study revealed the superiority of quetiapine and olanzapine over ziprasidone with remarkably less severe extrapyramidal adverse effects, especially with lower drop-out and treatment discontinuation. There were no differences in terms of other adverse events although the risk of treatment discontinuation was higher in female patients.

*Trial registration* 2012-3-88. Registered 20 July 2012

## Background

It has been recognized that first-episode schizophrenia represents a critical stage of illness during which the effectiveness of therapeutic interventions can affect long-term outcome [1, 2]. Antipsychotics (APs) are still a mainstay of treatment for schizophrenia and other psychotic disorders, although some evidence in recent years questioned their effects on long-term recovery [3–6]. Treating first-episode schizophrenic patients with reasonable medications according to the characteristics of the symptoms in individuals is particularly critical for patients’ continuous relief of symptoms and their social functional recovery. If the symptoms of first-episode schizophrenia did not remit within a comparatively shorter time with adequate treatment, there would be a considerably high risk of a poor long-term outcome, particularly for the patients with deterioration in premorbid social functioning [7].

Although some first-generation antipsychotics (FGAs) were still used in some countries, they were no longer widely used in recent years due to high rates of extrapyramidal side effects [8]. Second-generation antipsychotics (SGAs) have not yet shown significant advantages than first-generation antipsychotics (FGAs), although they have been extensively utilized as the first-line drug for treating patients with first-episode schizophrenia over the past years, as they can often cause weight gain and even alter lipid and glucose metabolism [9–11]. Some of the reviews have recently suggested that whether FGAs or SGAs are more effective is still inconclusive [12, 13]. Although no difference in the efficacy between atypical APs from many meta-analysis or systemic review reports, the differences in adverse effects were robust, such as tolerability, metabolic disorders, and safety [10, 14]. However, some research has revealed that there are no consistent differences in efficacy among atypical antipsychotics except for clozapine [15]. The differences in effectiveness, treatment discontinuation, and side effects among atypical antipsychotics still remain an interesting topic in clinical practice although the conventional clinical trials in the real world usually cannot provide a hierarchy based on pairwise comparison of antipsychotic efficacy or head-to-head clinical trials. The good response to initial antipsychotics, as well as the treatment intolerance or discontinuation and subsequent relapse, was obviously associated with a high remission rate and subsequent development of social functional disability in first-episode schizophrenia [2]. Whereas, in the CATIE study, up to 74% of patients interrupted their study drugs before 18 months, it is not surprising that high rates of discontinuation were common in some naturalistic studies in schizophrenic patients with a long exposure to antipsychotics [4]. A previous study has also indicated that it is unavailable to guide treatment decisions prior to medication response for treating first-episode schizophrenic patients [16].

Indeed, there is no well-defined guideline for clinicians to determine which kind of atypical APs is more effective or available for treating first-episode schizophrenia [17]. To our knowledge, there is relatively little information about the differences in effectiveness, acceptability, and safety between 5 kinds of atypical antipsychotics in the treatment of first-episode schizophrenia despite the existence of many meta-analyses or head-to-head study [18].

Properly balancing risks and benefits of antipsychotics, guaranteeing a better adherence to AP treatment, and achieving a better prognosis are the crucial factors and real challenges for the long-term clinical management of schizophrenia, especially in the treatment of first-episode schizophrenic patients. This study attempts to understand whether there are differences in efficacy, acceptability, and safety between the five kinds of atypical antipsychotics in the treatment of first-episode schizophrenia.

### Study methods

The study was carried out at the Huaian No. 3 People’s Hospital, Teaching Hospital of Xuzhou Medical University, Jiangsu Province. The subjects were selected from among the inpatients from October 2012 to November 2014. The protocol for the study was granted approval from the scientific and ethics committee of Huaian No. 3 People’s Hospital (Ethical Review No. HASYkjk 2012006). The patient or guardian signed the informed consent before the enrollment of the study.

### Study design

In the present study, an open-label and randomized controlled trial rooted in everyday clinical practice with a duration of at least 6–8 weeks was conducted. Patients were screened by clinicians and underwent a DSM-IV diagnosis of schizophrenia based on the Mini-International Neuropsychiatric Interview [19]. All eligible patients were randomized to one of the five kinds of APs (aripiprazole, risperidone, quetiapine, olanzapine, and ziprasidone), and the decision on the study drug to be used was based on the computer-generated random sequence after enrollment of the patients. Given the fact that the study was an open arm study, the decision on when and why the APs should be discontinued was mainly based on the clinical decision of the treating physician for all-cause, such as poor therapeutic effects or severe adverse events, or consultation with the treating team during the entire study period. No other specific strategies of maintaining or improving compliance were recommended during the study period. If poor therapeutic effects or no response or severe adverse events appeared after antipsychotic titration, the clinician would make timely decision to combine with another antipsychotic or discontinue the initially allocated antipsychotics 1–2 weeks before the initial treatment according to the predefined study protocol.

Switching to another antipsychotic would be considered as the treatment discontinuation due to no therapeutic effects during the study period. Clinicians were unobtrusively prompted to monitor adherence, and the reasons for treatment discontinuation were also collected as much as possible.

### Patient population

All patients did not receive any antipsychotics, mood stabilizers, antidepressants, or a system of psychological treatment before the enrollment of this study; for the purpose of reducing the influence of confounding factors, the patients with severe or unstable physical diseases and intolerance to antipsychotic treatment, and/or with age of onset ≥ 50 years, and/or the duration of illness ≥ 5 years were excluded from the present study; women with pregnancy or during lactation were not included.

**Fig. 1:**
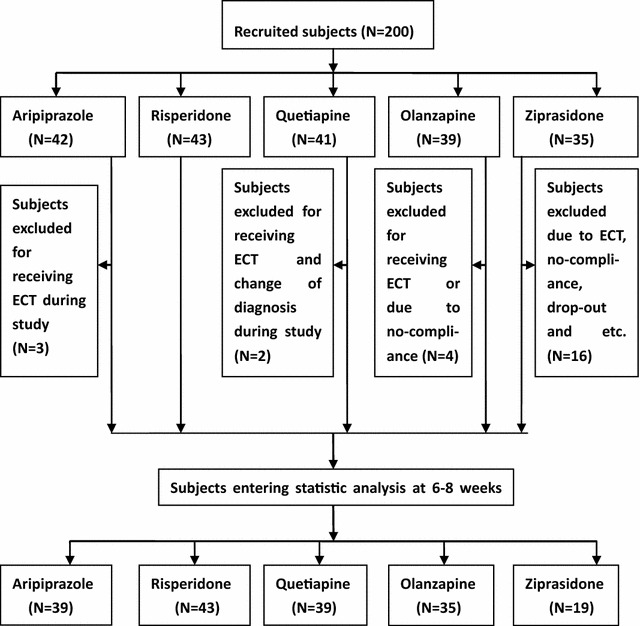
Diagram showing the study design and flow of subjects in the study

A total of 200 inpatients who met the above study criteria were enrolled in the study, but only 175 of the total 200 inpatients met the enrollment criteria were analyzed at the end of the study due to receiving electroconvulsive therapy (ECT), change of diagnosis, non-compliance, drop-out, and other reasons in some of the group (Fig. 1). Figure 1 shows the study design and flow of subjects in the study.

### Drug administration

Eligible patients were randomized to non-blind oral therapy with one of the following antipsychotics: aripiprazole, risperidone, quetiapine, olanzapine, and ziprasidone. And the initial dose was titrated gradually depending on the patients’ tolerability within the first or second week. If well tolerated, aripiprazole was increasingly titrated from the initial dose of 5 mg up to a maximum dose of 30 mg a day, and the mean (SD) dose of aripiprazole at study endpoint was 25.65 (6.45) mg/day; risperidone was titrated from the initial 0.5–1 mg up to a maximum of 6 mg a day, and the mean (SD) daily dose at study endpoint was 5 (2.51) mg; quetiapine was titrated from the initial 50 mg up to a maximum dose of 900 mg a day, and the mean (SD) dose of quetiapine at study endpoint was 634.52 (207.59) mg/day; olanzapine was titrated from the initial 5 mg up to a maximum dose of 20 mg a day, and its mean (SD) dose at study endpoint was 17.62 (3.4) mg/day; and ziprasidone was titrated from the initial 40 mg up to a maximum dose of 160 mg a day, and its mean (SD) daily dose at study endpoint was 102.86 (49.57) mg.

Temporary co-administration of benzodiazepines, anticholinergics, or beta-blockers should be considered for the management of the emerged side effects. Haloperidol injection 5–10 mg a day was sometimes used for a short period of time when necessary (e.g., the patient with aggression and/or more violent behavioral disorders, such as agitation or hostility).

### Assessment of clinical efficacy, acceptability, and safety

After antipsychotic titration, patients with concomitant antipsychotics or persistent usage of initially assigned drugs at the end of the study were considered to be effective cases and incorporated in the analysis. The psychopathological symptoms were rated at baseline and after 6–8 weeks of treatment using Brief Psychiatric Rating Scale, and the comparison of clinical efficacy between the five kinds of APs after the endpoint of the study (i.e., 6–8 weeks) was made by BPRS total scores’ deduction rate (baseline BPRS total scores − BPRS total scores post treatment/baseline BPRS total scores). The acceptability of each kind of antipsychotic was assessed by the discontinuation rate and the remaining use of the initially assigned medication rate at the end of the study. The safety of each antipsychotic at the end of the study was mainly evaluated by extrapyramidal symptoms, blood routine test, liver function test, and electrocardiogram (ECG) examination between the five kinds of antipsychotics. The assessment of BPRS scale was conducted by three psychiatric doctors trained with well consistency among them (correlation coefficient = 0.88–0.91).

### Statistical analysis

The statistical analysis was carried out using SPSS 13.0 (SPSS Inc., Chicago, Illinois, USA). The categorical variables were tested using a *χ* ^2^ test, whereas the continuous variables were tested by means of a paired *t* test before and after the study in every antipsychotic group, and variance analysis between the five kinds of antipsychotic groups was also conducted. The data of age of onset were transformed by base-e logarithm during statistical analysis due to the skewness distribution. The deduction rate of BPRS total scores at the end of the study between the five kinds of APs groups were compared by covariance analysis because of difference in age of onset. Binary logistic regression analysis was also conducted to assess the impact of risk factors on the treatment discontinuation of initially assigned APs at baseline.

All statistical tests were two tailed. Values are represented as mean ± standard deviation (SD). *P* value < 0.05 was considered statistically significant.

## Results

### Demographic and clinical features of study participants

No statistically significant differences of gender, the course of illness, and BPRS total scores before treatment were found among the five antipsychotic groups (*P* > 0.05) despite the fact that the patients in the ziprasidone group were younger than those in the other four antipsychotic groups, as shown in Table 1.

**Table 1:** Comparison of patients’ demographic and clinical features before treatment

Groups	*N*	Gender (M/F, *n*)	Age (years)	Marital status (M/U/D)	Duration of illness (months)	Baseline BPRS
Ari.	39	21/18	26.31 ± 8.09	16/22/1	10.29 ± 13.29	54.49 ± 10.46
Ris.	43	22/21	32.37 ± 8.45	26/15/2	10.34 ± 15.67	54.12 ± 10.21
Que.	39	18/21	28.77 ± 8.31	19/17/3	4.41 ± 8.01	56.72 ± 11.46
Ola.	35	17/18	25.54 ± 6.93	15/19/1	9.29 ± 13.22	57.40 ± 14.90
Zip.	19	3/16	22.26 ± 5.41	7/12/0	7.31 ± 12.96	57.79 ± 15.34
*χ* ^2^/*F*		8.49	7.38	8.36	1.45	0.65
*P* value		> 0.05	< 0.01	> 0.05	> 0.05	> 0.05

Values are represented as mean ± SD; *N*: number of patients; M/F: male/female; Ari.: aripiprazole; Ris.: risperidone; Que.: quetiapine; Ola.: olanzapine; Zip.: ziprasidone. M: married; U: unmarried; D: divorced

### Clinical efficacy between the five kinds of APs at the study endpoint

For the purpose of fully understanding the differences in efficacy between the five kinds of APs in first-episode schizophrenic patients, only 101 patients with continuous use of baseline APs (including the use of concomitant APs in each group) were analyzed at the endpoint of the study except for the patients with full treatment discontinuation of baseline APs. The BPRS total scores in each of the five kinds of the antipsychotic group at the end of the study were significantly decreased from the baseline (*P* < 0.01, Table 2). After controlling the impact of the covariable (i.e., the age of onset), the deduction rate of BPRS total scores at the end of the study in the risperidone group was still significantly higher than that in the aripiprazole (*P* < 0.01) and olanzapine groups (*P* < 0.05), whereas no statistically significant differences in the deduction rate of BPRS total scores were found between the other drug groups (*P* > 0.05, Table 2).

**Table 2:** Changes of BPRS total scores of patients who fully or partly maintained the initial treatment from baseline to endpoint among the five kinds of antipsychotic drugs

Groups	*N*	BPRS total score at baseline	BPRS total score at endpoint	Deduction rate of BPRS total score
Aripiprazole	23	54.00 ± 10.39	29.96 ± 9.05	0.44 ± 0.13
Risperidone	23	56.04 ± 10.39	24.87 ± 7.05	0.55 ± 0.12^ab^
Quetiapine	27	58.48 ± 11.16	28.74 ± 6.21	0.50 ± 0.11
Olanzapine	21	53.10 ± 11.5	26.90 ± 4.75	0.47 ± 0.15
Ziprasidone	7	58.71 ± 14.59	25.86 ± 4.91	0.54 ± 0.13
*F* value		0.97	1.95	2.97
*P* value		> 0.05	> 0.05	0.023

Deduction rate of BPRS score: (baseline BPRS total scores − post-treatment BPRS total scores)/baseline BPRS total scores. Values are represented as mean ± SD

*N*: number of patients

^a^Comparison of risperidone with aripiprazole, *P* < 0.01

^b^Comparison of risperidone with olanzapine, *P* < 0.05

### Comparison of acceptability between the five kinds of APs at the study endpoint

Only 101 cases of inpatients remained in the baseline antipsychotic and concomitant antipsychotic treatment except for 74 cases of inpatients with complete discontinuation of baseline antipsychotics after 6–8 weeks of treatment. The acceptability in 175 cases of inpatients was also compared between the five kinds of atypical APs at the endpoint of the study, as shown in Table 3. The comparison of acceptability was conducted by Chi-square test between the five kinds of APs (*χ* ^2^ = 16.51, *P* = 0.036). After further separating Chi-square test, there were significant differences between quetiapine (*χ* ^2^ = 5.46, *P* = 0.019), olanzapine (*χ* ^2^ = 5.6, *P* = 0.018), and ziprasidone in terms of the proportion of maintaining initially assigned antipsychotics. However, the difference in treatment discontinuation rate of the initially assigned APs at baseline was not found when comparing with the other two AP groups.

**Table 3:** Acceptability between the five kinds of antipsychotic groups after 6–8 weeks of treatment (*N*, %)

Groups	*N*	Disc. of initially assigned APs	Use of concomitant APs	Maintaining initially assigned APs
Aripiprazole	39	16 (0.41)	8 (0.21)	15 (0.38)
Risperidone	43	20 (0.47)	3 (0.069)	20 (0.47)
Quetiapine	39	12 (0.31)	4 (0.10)	23 (0.59)^a^
Olanzapine	35	14 (0.4)	0	21 (0.60)^a^
Ziprasidone	19	12 (0.63)	2 (0.11)	5 (0.26)

Values are represented as *N*: number of patients; APs: antipsychotics; Disc.: discontinuation

^a^Comparison of quetiapine and olanzapine with ziprasidone, both with *P* < 0.05

Binary logistic regression analysis was also carried out to assess the impact of multiple risk factors on the treatment discontinuation of initially assigned APs at baseline between the five kinds of atypical APs controlling for a number of confounding factors including the type of use of initially assigned APs at baseline, age of onset, gender, illness severity (BPRS total scores at baseline), and duration of illness as independent variables. The treatment discontinuation of initially assigned APs and maintaining the use of initially assigned APs (including patients with the use of concomitant APs and maintaining initially assigned APs until the study endpoint) were considered as the dependent variables. It was found that only the variable of gender was in the equation and was markedly related to treatment discontinuation of the initially assigned APs (regression coefficient *β*: 0.993, standard error: 0.32, Wald: 9.67, *P* value: 0.002). In addition, the drug use status was also further analyzed between male and female patients during the trial, and it was found that odds ratio of treatment discontinuation of the initially assigned APs in male and female patients was 0.37 (95% CI 0.198–0.693), which means that the risk of treatment discontinuation of the initially assigned antipsychotics in male patients was significantly lower than that in female patients (Table 4).

**Table 4:** Use status of the five kinds of antipsychotics between male and female patients after 6–8 weeks of treatment (*N*, %)

Gender	*N*	Disc. of initially assigned APs	Maintaining initially assigned and concomitant APs	*χ* ^2^ value	*P* value	OR for male, female
Male	81	24 (0.30)	57 (0.70)	9.90	0.002	0.37 (95% CI 0.198–0.693)
Female	94	50 (0.53)	44 (0.47)			

Values are represented as *N*: number of patients; APs: antipsychotics; Disc: discontinuation, OR: odds ratio

### Adverse events between the five kinds of APs at the study endpoint

Only 84 patients with the remaining use of initially assigned APs were compared at the end of the study in order to comprehensively understand adverse effects between the five kinds of atypical APs. Only difference in extrapyramidal symptoms (EPS) was found between five remaining use of initially assigned antipsychotics at the end of the study (*P* < 0.05, Table 5). Further analysis indicated that differences in EPS in the groups of aripiprazole, risperidone, and ziprasidone were significantly higher than that in the groups of quetiapine and olanzapine (*P* < 0.05–0.01). Other differences in abnormal ECG, liver function, constipation, and leucopenia were not detected between five remaining use of initially assigned antipsychotic groups (all *P* > 0.05, Table 5). Metabolic disturbance and sexual dysfunction were not analyzed due to incomplete data records and shorter study period in this study.

**Table 5:** Comparison of adverse events between the five kinds of antipsychotics after 6–8 weeks of treatment

Groups	*N*	EPS	Abnormal ECG	Abnormal liver function	Constipation	Leukopenia
Aripiprazole	15	6	4	5	1	0
Risperidone	20	6	4	6	2	0
Quetiapine	23	3	8	7	3	4
Olanzapine	21	1	8	5	1	2
Ziprasidone	5	3	3	1	0	0
*χ* ^2^		12.23	3.75	0.64	1.60	6.899
*P* value		< 0.05	> 0.05	> 0.05	> 0.05	> 0.05

Abnormal liver function: this mainly included elevated serum alanine aminotransferase (ALT) and aspartate aminotransferase (AST). ECG: electrocardiogram. Including S-T and T wave change, and prolongation of corrected Q-T interval

*N*: number of patients; EPS: extrapyramidal symptoms, including akathisia, acute dystonia, and parkinsonian syndromes

## Discussion

Current information about the choice of APs for the treatment of first-episode schizophrenia was relatively limited when an initially prescribed atypical antipsychotic treatment failed [20]. To our knowledge, the differences in effectiveness, acceptability, and safety between 2–3 kinds of second-generation antipsychotics in the treatment of first-episode schizophrenic patients mostly came from meta-analysis and some pairwise studies, but comparison between 5 different kinds of atypical antipsychotic drugs is rare.

This study showed all five kinds of atypical APs (aripiprazole, risperidone, quetiapine, olanzapine, and ziprasidone) have markedly clinical curative effectiveness in the treatment of first-episode drug-naïve schizophrenic patients. Whereas the deduction rate of BPRS total score at the endpoint of the study in the risperidone group was significantly higher than that in the aripiprazole and olanzapine groups, it was suggested that risperidone may be better than aripiprazole and olanzapine in terms of short-term clinical efficacy. This result was in line with the survey conclusions of the Expert Consensus Guideline, which showed that most clinicians chose risperidone as an alternative drug when olanzapine at an appropriate dosage fails to produce sufficient response [21, 22]. This is also consistent with the results of Leucht’s research [14], but inconsistent with reports from Krzystanek’s meta-analysis, which showed that the curative effect of olanzapine was remarkably better than those of some other second-generation antipsychotics, i.e., aripiprazole, risperidone, quetiapine, and ziprasidone, except for clozapine and amisulpride [23].

The comparison of acceptability between the five kinds of atypical APs revealed that quetiapine and olanzapine were significantly better than ziprasidone after the endpoint of the study, especially in terms of maintaining the use of the initially assigned APs, whereas the differences between other atypical APs were not found. This might be associated with fewer extrapyramidal symptoms in patients with quetiapine and olanzapine treatment, even at higher doses. In contrast, ziprasidone often has relatively more extrapyramidal symptoms, especially when used at higher doses [24]. This is completely consistent with the result of Roussidis et al. [1] and partially consistent with the results of Leucht’s research [14], but inconsistent with the research by Jin et al. [25] who suggested that treating clinicians often tended to exclude olanzapine and prefer aripiprazole or ziprasidone as one of the possible choices in patients with metabolic problems. In fact, patients with aggression, excessive excitement, or noisy, rather than metabolic problems, were often needed to be controlled rapidly using concomitant antipsychotic. This concurred with the reports by Edlinger and Cotton et al. [26, 27] who concluded that olanzapine has desirable effects during acute inpatient treatment despite the sedative effects. Other reasons for this inconsistency might be related to the inconsistent criteria in terms of judging acceptability of study drugs and relatively fewer patients enrolled in the ziprasidone group in this study. Crespo-Facorro et al. reported that first-episode schizophrenic patients treated with quetiapine had a higher risk of treatment discontinuation than those treated with aripiprazole and ziprasidone at midterm due to insufficient effectiveness [28]. However, no significant differences were found in treatment discontinuation of the initially assigned APs between the five APs in the present study; the possible reason might be that the mean dose of quetiapine used may not be enough for treating first-episode schizophrenic patients. In addition, gender was markedly related to treatment discontinuation of the initially assigned APs, and the risk of treatment discontinuation in male patients was significantly lower than that in female patients. It might be related to intolerability that occurred easily or more concerns on discomfort in female patients.

The National Institute for Clinical Excellence (NICE) [19] suggested choosing an antipsychotic with best efficacy and tolerability for an individual is more appropriate than the drug category [18]. The differences in extrapyramidal symptoms (EPS) during this trial were consistent with some previous reports [18, 29]. Quetiapine and olanzapine with less EPS displayed high-level therapeutic adherence and showed great significance for successful treatment of first-episode schizophrenic patients. Whereas the side effects of weight gain and metabolic disturbance are much more concerned in recent years, these disadvantages should not be ignored.

In summary, all five atypical antipsychotics (aripiprazole, risperidone, quetiapine, olanzapine, and ziprasidone) had markedly curative effects on first-episode drug-naïve schizophrenic patients. Risperidone showed better antipsychotic effects than aripiprazole and olanzapine in this study. The acceptability of quetiapine and olanzapine was remarkably better than that of ziprasidone, especially regarding maintaining the use of initially allocated APs. The risk of treatment discontinuation was higher in female patients. The differences in EPS were commonly observed in the treatment of aripiprazole, risperidone, and ziprasidone although other adverse effects were similar.

## Limitations of the study

This was an open-label trial that was prone to bias when conclusions to be made. In addition, what needs to be further explained was that the efficacy was often difficulty to be judged if the use of augmentation was taken, even it is attributed to the sole antipsychotic treatment. The associated problems, such as metabolic disorder and sexual dysfunction, were not analyzed because of incomplete records and design of the trial. In addition, the number of patients enrolled in the ziprasidone group was somewhat smaller due to non-compliance, receiving ECT, and drop-out. And the reasons for treatment discontinuation in each antipsychotic group were not fully recorded and analyzed. Large, well-designed trials are warranted to gain more information about the therapeutic effects, acceptability, and adverse effects of different atypical antipsychotic drugs.

## Abbreviations

BPRS: Brief Psychiatric Rating Scale
APs: antipsychotics
FGAs: first-generation antipsychotics
SGAs: second-generation antipsychotics
CATIE: Clinical Antipsychotic Trials of Intervention Effectiveness
DSM-IV: the Diagnostic and Statistical Manual of Mental Disorders, Fourth Edition
ECT: electroconvulsive therapy
SD: standard deviation
OR: odds ratio
ECG: electrocardiogram
EPS: extrapyramidal symptoms

## References

[1] Roussidis A, Kalkavoura C, Dimelis D, Theodorou A, Ioannidou I, Mellos E (2013). Reasons and clinical outcomes of antipsychotic treatment switch in outpatients with schizophrenia in real-life clinical settings: the ETOS observational study. Ann Gen Psychiatry.

[2] Salimi K, Jarskog LF, Lieberman JA (2009). Antipsychotic drugs for first-episode schizophrenia: a comparative review. CNS Drugs.

[3] Dunayevich E, Ascher-Svanum H, Zhao F, Jacobson JG, Phillips GA, Dellva MA (2007). Longer time to antipsychotic treatment discontinuation for any cause is associated with better functional outcomes for patients with schizophrenia, schizophreniform disorder, or schizoaffective disorder. J Clin Psychiatry.

[4] Lieberman JA, Stroup TS, McEvoy JP, Swartz MS, Rosenheck RA, Perkins DO (2005). Effectiveness of antipsychotic drugs in patients with chronic schizophrenia. N Engl J Med.

[5] Samaha AN, Seeman P, Stewart J, Rajabi H, Kapur S (2007). “Breakthrough” dopamine supersensitivity during ongoing antipsychotic treatment leads to treatment failure over time. J Neurosci..

[6] Amato D, Natesan S, Yavich L, Kapur S, Müller CP (2011). Dynamic regulation of dopamine and serotonin responses to salient stimuli during chronic haloperidol treatment. Int J Neuropsychopharmacol..

[7] Friis S, Melle I, Johannessen JO, Røssberg JI, Barder HE, Evensen JH (2015). Early predictors of ten-year course in first-episode psychosis. Psychiatr Serv..

[8] Miyamoto S, Duncan GE, Marx CE, Lieberman JA (2005). Treatments for schizophrenia: a critical review of pharmacology and mechanisms of action of antipsychotic drugs. Mol Psychiatry.

[9] Wang CJ, Zhang ZJ, Sun J, Zhang XB, Mou XD, Zhang XR (2006). Serum free fatty acids and glucose metabolism, insulin resistance in schizophrenia with chronic antipsychotics. Biol Psychiatry.

[10] Newcomer JW (2007). Antipsychotic medications: metabolic and cardiovascular risk. J Clin Psychiatry.

[11] Leucht S, Corves C, Arbter D, Engel RR, Li C, Davis JM (2009). Second-generation versus first-generation antipsychotic drugs for schizophrenia: a meta-analysis. Lancet.

[12] Cheng F, Jones PB (2013). Drug treatments for schizophrenia: pragmatism in trial design shows lack of progress in drug design. Epidemiol Psychiatr Sci..

[13] Crespo-Facorro B, Perez-Iglesias R, Mata I, Martinez-Garcia O, Ortiz V, Pelayo-Teran JM (2012). Longterm (3-year) effectiveness of haloperidol, risperidone and olanzapine: results of a randomized, flexible-dose, open-label comparison in first-episode nonaffective psychosis. Psychopharmacology (Berlin)..

[14] Leucht S, Cipriani A, Spineli L, Mavridis D, Orey D, Richter F (2013). Comparative efficacy and tolerability of 15 antipsychotic drugs in schizophrenia: a multiple-treatments meta-analysis. Lancet.

[15] Tandon R, Belmaker RH, GattazWF Lopez-Ibor JJ, Okasha A, Singh B (2008). World Psychiatric Association Pharmacopsychiatry section statement on comparative effectiveness of antipsychotics in the treatment of schizophrenia. Schizophr Res.

[16] Robinson DG, Gallego JA, John M, Petrides G, Hassoun Y, Zhang JP (2015). A randomized comparison of aripiprazole and risperidone for the acute treatment of first-episode schizophrenia and related disorders: 3-month outcomes. Schizophr Bull.

[17] Thomas SP, Nandhra HS, Singh SP (2012). Pharmacologic treatment of first-episode schizophrenia: a review of the literature. Prim Care Companion CNS Disord..

[18] Zhu Y, Krause M, Huhn M, Rothe P, Schneider-Thoma J, Chaimani A (2017). Antipsychotic drugs for the acute treatment of patients with a first episode of schizophrenia: a systematic review with pairwise and network meta-analyses. Lancet Psychiatry..

[19] Sheehan DV, Lecrubier Y, Sheehan KH, Amorim P, Janavs J, Weiller E (1998). The Mini-International Neuropsychiatric Interview (M.I.N.I.): the development and validation of a structured diagnostic psychiatric interview for DSM-IV and ICD-10. J Clin Psychiatry.

[20] Takahashi H, Yoshida K, Ishigooka J, Higuchi H (2006). Switching to olanzapine after unsuccessful treatment with risperidone during the first episode of schizophrenia: an open-label trial. J Clin Psychiatry.

[21] Takahashi H, Yoshida K, Ishigooka J, Higuchi H (2006). Switching to risperidone after unsuccessful treatment of olanzapine in the first-episode schizophrenia: an open trial. Prog Neuro-psychopharmacol Biol Psychiatry.

[22] Li H, Luo J, Wang C, Xie S, Xu X, Wang X (2014). Efficacy and safety of aripiprazole in Chinese Han schizophrenia subjects: a randomized, double-blind, active parallel-controlled, multicenter clinical trial. Schizophr Res..

[23] Krzystanek M, Krupka-Matuszczyk I (2011). An open, large, 6-month naturalistic study of outcome in schizophrenic outpatients, treated with olanzapine. Hum Psychopharmacol..

[24] Li YM, Zhao JP, Ou JJ, Wu RR (2012). Efficacy and tolerability of ziprasidone vs. olanzapine in naive first-episode schizophrenia: a 6-week, randomized, open-label, flexible-dose study. Pharmacopsychiatry..

[25] Jin H, Shih PA, Golshan S, Mudaliar S, Henry R, Glorioso DK (2013). Comparison of longer-term safety and effectiveness of 4 atypical antipsychotics in patients over age 40: a trial using equipoise-stratified randomization. J Clin Psychiatry.

[26] Edlinger M, Rettenbacher MA, Kemmler G, Biedermann F, Widschwendter CG, Fleischhacker WW (2016). Prescribing practice in inpatients versus outpatients with schizophrenia initiating treatment with second-generation antipsychotics: a naturalistic follow-up study. J Clin Psychopharmacol.

[27] Cotton MA, Johnson S, Bindman J, Sandor A, White IR, Thornicroft G, Hoult J, McKenzie N, Bebbington P (2007). An investigation of factors associated with psychiatric hospital admission despite the presence of crisis resolution teams. BMC Psychiatry..

[28] Crespo-Facorro B, de la Foz VO, Mata I, Ayesa-Arriola R, Suarez-Pinilla P, Valdizan EM (2014). Treatment of first-episode non-affective psychosis: a randomized comparison of aripiprazole, quetiapine and ziprasidone over 1 year. Psychopharmacology.

[29] Crespo-Facorro B, Pérez-Iglesias R, Mata I, Ortiz-Garcia de la Foz V, Martínez-Garcia O (2013). Aripiprazole, ziprasidone, and quetiapine in the treatment of first-episode nonaffective psychosis: results of a 6-week, randomized, flexible-dose, open-label comparison. J Clin Psychopharmacol.

